# Prevalence and associated risk factors of chronic kidney disease: A case study within SIME clinics in Quito, Ecuador 2019–2021

**DOI:** 10.3389/fmed.2022.908551

**Published:** 2022-08-19

**Authors:** Lucía Eguiguren-Jiménez, Joshua Miles, Jaime Ocampo, Jeanette Mary Andrade

**Affiliations:** ^1^Food Science and Human Nutrition Department, University of Florida, Gainesville, FL, United States; ^2^Statistics Department, University of Florida, Gainesville, FL, United States; ^3^School of Public Health, San Francisco de Quito University, Quito, Ecuador

**Keywords:** chronic kidney disease, prevalence, blood pressure, blood glucose, eGFR

## Abstract

**Background:**

Ecuador has been experiencing an epidemiological transition due to its demographic and lifestyle changes, where non-communicable diseases are the leading cause of death, including chronic kidney disease (CKD). Quito, Ecuador's capital city, is one of the cities burdened by CKD, yet it is unknown the factors that contribute to the rising incidence of this disease. The purpose of this study was to estimate the prevalence of CKD among non-institutionalized adults in Quito between 2019 and 2021, and to examine its associations with various risk factors.

**Methods:**

For the analysis of prevalence, the *Kidney Disease: Improving Global Outcomes* guidelines were used, where an estimated glomerular filtration rate (eGFR) of < 60 ml/min/1.73 m^2^ was counted as a presumed case of CKD. The Chronic Kidney Disease Epidemiology Collaboration (CKD-EPI) equation was used to calculate eGFR. Multiple linear regression models were used to determined associations between blood pressure, blood glucose, sex, and zone with eGFR. A *t-*test of independence was used to determine difference in means between sex and zone and eGFR.

**Results:**

A prevalence of 7.2% was found, in which almost 45% of the participants were classified within stages 2–4 of this disease. The risk factors that were significantly associated with eGFR were systolic blood pressure (β = −0.43, *p* < 0.001), sex, and zone (*p* < 0.001).

**Conclusions:**

Overall a high prevalence of CKD was found among adults who visited SIME clinics in Quito. Associations between main risk factors and eGFR were found, yet further research is needed to explore CKD in Ecuador and its main cities.

## Introduction

Chronic kidney disease (CKD) is a major public health concern with its rising prevalence and higher mortality rates (1). Globally, the prevalence of CKD is 9.1% or about 700 million people, in which females have a higher prevalence (9.5%) compared to males (7.8%), and a mortality rate of 4.6% (2). CKD has five different stages based on the estimated Glomerular Filtration Rate (eGFR), where CKD is presumed with an eGFR of <60 ml/min/1.73 m^2^ (3). eGFR is considered the best overall indicator of kidney function (4) and is determined through the utilization of serum creatinine and characteristics of the patient such as sex, race and age (5). The Modification of Diet in Renal Disease (MDRD) and the Chronic Kidney Disease Epidemiology Collaboration (CKD-EPI) are used globally to calculate the eGFR. A meta-analysis compared eGFR from adults with CKD based on the CKD-EPI equation to the MDRD equation. Results showed that the CKD-EPI equation had less bias when classifying different populations, greater precision and accuracy considering the demographic profiles of participants (e.g., age, sex, race), in comparison to the MDRD equation (6). Furthermore, Levey et.al discovered that the MDRD equation led to higher prevalence estimates due to its imprecision when detecting higher values of GFR (4). Meanwhile, the Kidney Disease: Improving Global Outcomes (KDIGO) guidelines state that in the absence of specific modifications such as race, ethnicity, or regional differences, it is acceptable to use the CKD-EPI equation for determining eGFR (3).

The progression of this disease can be attributed to different risk factors such as elevated blood glucose and high blood pressure (2). Studies demonstrated that blood pressure levels below 130/90 mmHg reduces the risk for CKD, cardiovascular disease, and others whereas above 130/90 mmHg increases that risk (7–10). Nonetheless, it is worth noting that the systolic and diastolic blood pressure, may be equally and individually important to predict the risk for CKD. A few studies have shown that systolic blood pressure (SBP) is highly associated with adverse kidney outcomes, but not diastolic blood pressure (DBP) (11, 12). Moreover, elevated blood glucose levels (i.e., hyperglycemia) have been known to disrupt kidney's function and lead to impairment of glucose homeostasis (13). When hyperglycemia is present, there are several consequences that progressively damage the structure of the kidney, such as a mesangial expansion of the matrix and thickening of the glomerular basement membrane, which are known to cause an increase of the systemic pressure and elevated excretion of protein in urine (i.e., proteinuria), resulting in a reduction of glomerular filtration (14). Furthermore, when diabetic nephropathy is present, hemodynamic modifications occurs, which changes normal renal blood flow and causes the kidney to increase glomerular filtration, triggering not only an alteration of kidney's functions but also affecting the body homeostasis overall (14).

CKD is greatly affecting Latin American countries, especially Ecuador (15) in which CKD has increased by more than 50% of the disability-adjusted life years (DALYs) rate from 1990 to 2017 (2). Few studies, though, have focused on risk factors and prevalence of certain regions within Ecuador. One study performed in Cuenca, Ecuador, revealed the prevalence of CKD was at 10.6% with the highest prevalence observed in individuals younger than 65 years of age, and a higher prevalence of CKD detected in urban zones (16). Even though this is one of the few prevalence studies performed in Ecuador, the results are based on the MDRD equation, thus caution must be considered when comparing to similar studies using the CKD-EPI equation.

One specific region in Ecuador where CKD is the leading cause of mortality is Quito. Quito, the capital of Ecuador, is located within the province Pichincha. Compared to other cities in Pichincha, it is a densely populated area with 2.6 million people, who are predominately female (60.5%), are between the ages of 20–39 years (34%) and considered Mestizos (American India and White, 82.8%) (17). Even though mortality rates from CKD are high in this region (18), limited information is available with the prevalence and risk factors associated with this disease in Quito. Therefore, this study aimed to explore CKD prevalence and the impact that different risk factors such as blood glucose and blood pressure, and demographics have on estimated glomerular filtration rate (eGFR) among adults residing in Quito.

## Materials and methods

### Study design

A retrospective cross-sectional case study was conducted among non-institutionalized adults who visited Sistemas Médicos (SIME) clinics in Quito, for routine physical exams during the years of 2019–2021. Sistemas Médicos (SIME), is a group of clinics that offers primary health care attention. SIME clinics are found within the main parishes of Quito. Two of these clinics are in rural zones: Carapungo-Calderón and Los Chillos, while the other two are in urban zones: Cumbayá and La Carolina. SIME clinics have a commitment to the community to reduce and prevent the prevalence of non-communicable diseases through research and medical advancements (19). This study was approved by the Ethics and Research Committee of Human subjects of CEISH-USFQ (IE02-E158-2021-CEISH-USFQ) and by the University of Florida Institute of Review Board (IRB202101202) as exempt.

### Study population

**Figure 1:**
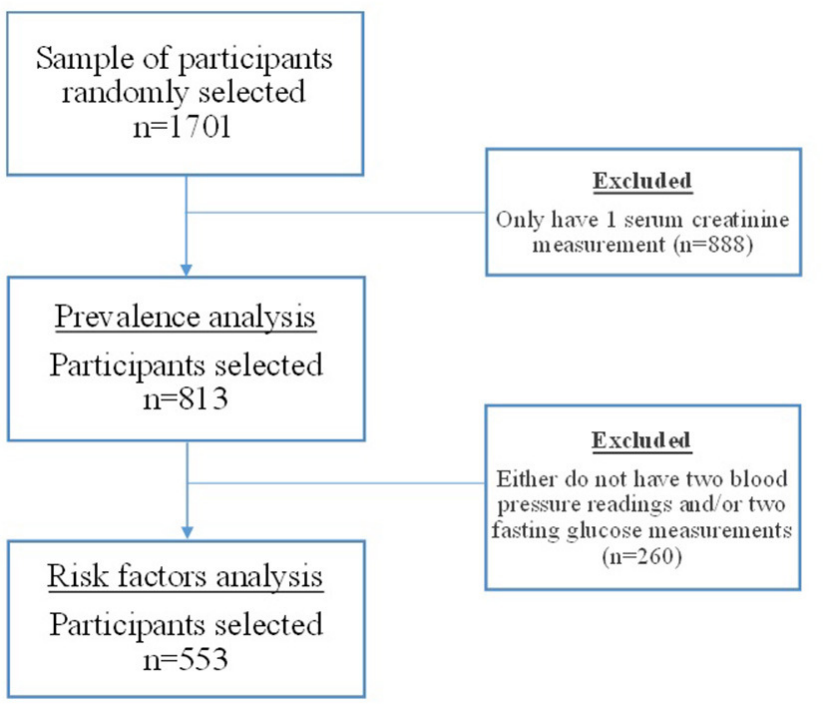
Flow diagram of the selection process for the sample size.

For this study, the inclusion criteria comprised: (1) adults over 18 years of age, (2) demographic data (sex and age), (3) at least two readings of blood pressure, (4) at least two measures of blood glucose, and (5) at least two measures of serum creatinine. The criteria followed World Health Organization (WHO) (20, 21), and KDIGO guidelines, for detection of presumed hypertension, diabetes and CKD cases, respectively, in which the blood pressure readings, blood glucose and serum creatinine measurements were mean averaged for analysis. These readings and measurements may have occurred at different time points for each participant (e.g., collection of all markers within the same year or every other year). Participants were excluded if they did not meet the above criteria and/or if they were pregnant/lactating women. The de-identified data was sent to one of the researchers (L.E.-J.), who collected and organized the information in a matrix previously created by the researchers (L.E.-J., J.M.A). The dataset included 17,000 participants from which 1,701 participants (10%) were randomly selected following the methodology of another population-based study (22). The primary outcome in this study was eGFR. Based on the sample of 1,701 participants, 813 met the study inclusion criteria to analyze CKD prevalence, while 553 met the criteria for all the variables of interest. Assuming the current prevalence of 11.3% among the entire Ecuadorian population (2) and assuming an incidence of 5% within the study group, this would be a 95% power with a precision of 6.3–16.3% to detect incidence of CKD. See Figure 1 for a flow diagram of the selection process for the sample size of this study.

### CKD-EPI equation

The eGFR was determined based on the updated 2022 CKD-EPI equation to determine CKD prevalence. The CKD-EPI equation can be seen below:


$$\begin{array}{lll} eGFR & = & \;142\;*{\left(minstandardized\frac{Scr}{K},\;1\right)}^{\alpha }\;*\\ & & {\left(maxstandardized\frac{Scr}{K},\;1\right)}^{-1.200}\;*\;0.9938^{Age}\;*\;1.012\\ & & \left[if\;female\right] \end{array}$$

where Scr is serum creatinine, *K* is 0.7 for females and 0.9 for males, α is−0.241 for females and - 0.302 for males, min indicates the minimum of *Scr/K* or 1, and max indicates the maximum of *Scr/K* or 1.

### Statistical analysis

Statistical analyses were carried out using the statistical program R version 4.1.2 (23, 24). Descriptive analyses were used to represent the distribution of the sample by different variables such as sex, rural or urban zones, while age, blood glucose, and blood pressure, and serum creatinine were presented as mean ± standard deviation (SD). To identify the prevalence of CKD, a dichotomous eGFR variable (<60 vs. ≥60 ml/min/1.73 m^2^) was used to determine the presence of CKD according to the definition of KDIGO guidelines (3) where an eGFR < 60 ml/min/1.73 m^2^ was counted as a CKD case. Additionally, based on the eGFR, participants were categorized into the five different stages of CKD to determine frequencies. To examine the impact of blood glucose and blood pressure on eGFR values, a multiple linear regression model was used. A *t*-test was used to determine differences between sex and zone on eGFR. Statistical significance was determined at *p* < 0.05.

## Results

### Characteristics of the samples

For the analysis of CKD prevalence, the majority of participants were females (61.1%), all identified as Mestizo race, had an average age range between 18 and 44 years, and more participants visited urban zone clinics (85.6%) compared to those who visited the clinics located at rural zones (14.4%). Similarly, for the analysis of CKD risk factors, most of the participants were females (59.5%), the majority were less than 65 years of age, and more participants visited urban zones clinics (84.4%). Furthermore, 11.9% of participants had a mean systolic blood pressure above 140 mmHg, and 4.2% had mean values higher than 90 mmHg in diastolic blood pressure. The percentage of participants that presented mean values of fasting blood glucose above 126 mg/dl, was 3.8%, while presumed cases of CKD (<60 ml/min/1.73 m2) accounted for 7.2% of the sample (Table 1).

**Table 1:** Demographic characteristics.

**Group**	**N**	**Percentage (%)**
Sex (*n =* 813^*^)		
Females	497	61.1
Males	316	38.9
Zone (*n =* 813)		
Urbana	696	85.6
Rural	117	14.4
Age (*n =* 813)		
18–44	334	41.1
45–64	287	35.3
> 64	192	23.6
CKD (*n =* 813)		
eGFR <60 ml/min/1.73m^2^	58	7.2
eGFR ≥ 60 ml/min/1.73m^2^	755	92.8
Systolic blood pressure (*n =* 553^**^)		
<140 mmHg	487	88.1
≥140 mmHg	66	11.9
Diastolic blood pressure (*n =* 553^**^)		
<90 mmHg	530	95.8
≥90 mmHg	23	4.2
Blood Glucose (*n =* 553^**^)		
<126 mg/dl	532	96.2
≥126 mg/dl	21	3.8

* Participants that met inclusion criteria for: age, sex, zone, and 2 serum creatinine measurements.

** Participants that met inclusion criteria for: age, sex, zone, 2 serum creatinine, 2 blood pressure and 2 blood glucose measurements.

### CKD prevalence

The prevalence of CKD was 7.2% and almost 45% of the participants were classified within stages 2–4 (Table 2). Furthermore, the prevalence of CKD among those who visited an urban zone clinic compared to a rural zone clinic was determined at 6.62 and 1.02%, respectively. Prevalence of CKD among males was at 7.9% and among females was at 6.6%. Chi-square analysis revealed no significant correlations between rural and urban zones and eGFR, *X*^2^, (1, *N* = 813) = 1.49, *p* = 0.221; and between sex and eGFR, *X*^2^, (1, *N* = 813) = 0.30, *p* = 0.584.

**Table 2:** Prevalence of CKD by stage.

**Stage (eGFR ml/min/1.73m^2^)**	**CKD-EPI (N)**	**Prevalence Percentage (CKD-EPI) [%]**
Stage 1 (≥90)	455	55.9
Stage 2 (60–89.9)	300	36.9
Stage 3 (30–59.9)	56	6.9
Stage 4 (15–29.9)	2	0.3
Stage 5 (<15)	0	0

### Risk factors associated with eGFR

A multiple linear regression analysis showed that systolic blood pressure (β = −0.43, *p* < 0.001) and blood glucose (β = −0.09, *p* = 0.024) was negatively associated with eGFR (model 1) (Table 3). A second multiple regression model adding sex and zone as covariates showed that there was a negative association between sex (β = −3.87, *p* = 0.027), zone (β = −11.89, *p* < 0.001) and eGFR. However, the blood glucose variable was not significant in model 2 (β = −0.06511, *p* = 0.107) (Table 4). Furthermore by performing a *t*-test for independence, it was found that males had a lower eGFR than females, t_(731.2)_ = 4.71, *p* < 0.001, and adults who had visited rural zone clinics had lower eGFR compared to those who visited urban zone clinics, t_(174.1)_ = −6.56, *p* < 0.001.

**Table 3:** Factors associated with eGFR (Model 1).

**Coefficient**	**Estimate (95% CI)**	**SE**	**t. value**	**Pr(>|t|)**
(Intercept)	145.51 (129.853, 161.161)	7.97	18.26	<0.01^**^
Systolic blood pressure	−0.426 (-0.584,−0.267)	0.08	−5.27	<0.01^**^
Diastolic blood pressure	0.082 (-0.171, 0.335)	0.13	0.64	0.53
Blood Glucose	−0.092 (-0.173,−0.012)	0.04	−2.25	0.03

Multiple linear regression (Model 1): R2 = 0.09, R2 adjusted = 0.09, p < 0.001;

** p-value < 0.01.

**Table 4:** Factors associated with eGFR (Model 2).

**Coefficient**	**Estimate (95% CI)**	**SE**	**t.value**	**Pr(>|t|)**
(Intercept)	156.41 (140.68, 172.14)	8.01	19.53	<0.01^**^
Systolic blood pressure	−0.43 (-0.58,−0.27)	0.08	−5.43	<0.01^**^
Diastolic blood pressure	0.16 (-0.09, 0.42)	0.13	1.24	0.22
Blood Glucose	−0.07 (-0.14, 0.01)	−0.04	−1.61	0.11
Sex	−3.87 (-7.30,−0.44)	1.75	−2.22	0.03^*^
Zone	−11.89 (-16.29,−7.49)	2.24	−5.30	<0.01^*^

Multiple linear regression (Model 2): R2 = 0.14, R2 adjusted = 0.14, p < 0.001;

** p-value < 0.01;

* p-value < 0.05.

## Discussion

In this study, a prevalence of 7.2% was found among adults who visited SIME clinics. The majority of participants were classified within stages 1 and 2 of this disease following the KDIGO guideline criteria. The sample was best characterized as participants below 65 years of age, most of them women, and who visited more urban zone clinics than rural zone clinics. The risk factors that were significantly associated to eGFR, based on multiple regressions, were systolic blood pressure, blood glucose, sex, and zone.

CKD is a progressive disease in which detection can be a challenge since symptoms do not start until later stages of this disease, 3–5 (25), hence obtaining accurate information about the prevalence of this disease is fundamental to reduce the rates. In 2017, the Global, regional, and national burden of chronic kidney disease study showed that Ecuador's prevalence was 11.3% (2), which differs from this study where the prevalence was 7.2%. This report indicated to have limitations, one of them, was the use of predictive statistics to obtain prevalence estimates for the countries that lacked information about the incidence and prevalence of CKD, like Ecuador (2). Furthermore, classification of the eGFR through the different stages of CKD has been also considered an important barrier to determine accurate prevalence in different countries, especially considering the use of different equations such as CKD-EPI and MDRD to calculate eGFR (1). In congruence with this study, another study (26) showed that the prevalence of CKD stages 3-5 in low to middle income countries ranged from 7.4 to 13.1% with a median of 10.7%. Furthermore, a study conducted in Nicaragua among 1,242 participants showed that the prevalence of CKD was 5.3% and was most prevalent in those who were older, self-reported diagnosis of hypertension or diabetes (27). In a similar study done in another major city of Ecuador (16), the percentage of prevalence was 10.6%, using the MDRD equation, thus to truly compare prevalence and identify the severity of CKD, it is necessary for further studies to use one equation.

While detecting CKD is fundamental, understanding its main risk factors is equally important due to their impact on the progression of the disease. Hypertension is one of the leading causes of CKD, and in developing countries, it is attributed to be the cause of 21% of CKD cases (28). Yet, in Ecuador, and its main cities, accurate information elucidating the association between these chronic diseases is scarce. In this study, blood pressure components [Systolic Blood Pressure (SBP) and Diastolic Blood Pressure (DBP)] were analyzed individually, and the results revealed that SBP was negatively associated with eGFR, while DBP showed no significance. Similar to these results, the main findings of an observational retrospective study that included 1,323 individuals, indicated that time updated SBP was associated with CKD progression. Time-updated in that study referred to an average SBP at different time points. Furthermore, that study revealed that hazard ratios were higher among adults with SBP between 130 and 139 mmHg and above 140 mmHg (HR: 1.48 and 2.53 respectively), compared to the reference group (e.g., < 120 mmHg). Indicating that the higher SBP contributed to CKD. Meanwhile, DBP did not show a significant association with CKD progression (29). A possible explanation for this is the stiffness of arteries caused by high blood pressure, in which there is a return of blood pressure flowing back to the heart that increases the pressure of the systole compared to the diastole (30). Moreover, high blood pressure, in general, will cause a progressive loss of kidney function by injuring the afferent arteriole of the kidney, in which its ability to constrict and dilate is compromised, thus affecting the kidney's ability to remove waste and reduce GFR (31).

Although blood glucose was not significant after the addition of sex and zone as covariates in the second regression model, there remained a negative trend, in which a higher blood glucose will lower eGFR levels. Moreover, blood glucose is one of the main contributors of CKD and the prevalence of diabetes is on the rise in the Ecuadorian population (32) and one of the main causes of mortality in Quito (18). In the initial regression model, blood glucose had a statistically significant negative association with eGFR. Similar results were found in a descriptive study of 40 diabetic patients, in which they observed a decrease in GFR when glucose levels were reduced. In that study GFR was calculated through the creatinine clearance formula, which used creatinine from blood and urine samples. Through this equation, a normal range of GFR is between 80 and 130 ml/min, thus a decrease in GFR was considered a positive result. Furthermore, that study also demonstrated a reduction in hyperfiltration when glucose levels were reduced, which indicates a possible improvement of kidneys' function (33). These results could be explained by the kidneys' function to re-absorb glucose when needed, thus high glucose levels will enhance the kidney's absorption and filtration, causing microvascular complications, in which the kidney starts filtrating glucose and/or other large molecules, thus contributing to kidney disease (34).

In addition to the findings with blood glucose and SBP, sex and zone also were associated with eGFR. These findings are comparable to another population-based CKD prevalence study that was completed in Nicaragua, where males had higher odds (POR = 3.47) of CKD compared to females and those living in a rural zone was associated with an increased odds (POR = 2.10) of CKD. Even though this Nicaragua study did not compare risk factors with eGFR values directly, but with presumed cases of CKD, they followed the criteria for counting a CKD case at an eGFR <60 ml/min/1.73 m^3^, which means that zone and sex were associated with decreased eGFR values (22). Another study conducted among agricultural workers in El Salvador, obtained similar results, in which two rural communities had decreased eGFR (< 60 ml/min/1.73 m^3^) compared to urban communities. Furthermore, males had a significantly higher prevalence of decreased eGFR than females. The authors suggested that a decreased eGFR in males was due to their occupation. Regarding the difference between rural and urban zones, the authors explained that the studied communities were exposed to different climates and altitudes. In those residing in warmer climates, there may have been slight dehydration, contributing to the reduction in eGFR (35). In this study, even though the climates and altitudes were similar between rural and urban areas, individuals residing in the rural areas had lower mean eGFR compared to those residing in the urban areas. This difference might be explained by reduced access to health care centers, lack of nephrologists, and dialysis centers located in rural areas compared to urban zones (36). Concerning a lower mean eGFR in males compared to females, a probable justification might be that in Ecuador the majority of registered works are classified within the group of agriculture, livestock, forestry and fishing (37), where all of them are consider hard labor work, which might be related to dehydration issues and could possibly lead to decrease of kidney function as well.

### Limitations and strengths

To the best of the authors' knowledge, this is the first retrospective study that explored CKD prevalence and the associations between different risk factors and eGFR among adults who visited different clinic centers in Quito. Moreover, the aims of this study aligned with different goals set by a group of researchers as an action plan for determining CKD, which provided specific activities that can improve CKD monitoring and detection, highlighting the importance of surveillance systems within a country (1). However, this study does have limitations. First, more than half of the participants had to be excluded due to lacking at least two measurements of serum creatinine to calculate eGFR values. This indicates that serum creatinine is not part of basic blood work, even though the prevalence of CKD was high. Also, the lack of other laboratory markers that could help in the identification of proteinuria, is an important limitation. Second, for the analysis of risk factors, more participants were excluded due to lacking either blood glucose measurements and/or blood pressure readings, which could impact the results, especially considering the change in significance of blood glucose on eGFR. Also, the procedures used for the collection of serum creatinine, blood pressure and blood glucose, is unknown to the authors. Furthermore, no time series or method to assess progression of CKD prevalence in the population could be conducted as the time for collection of these measurements varied. Third, results cannot be comparable to other studies performed in Ecuador or Ecuador's main cities that can further contribute to elucidate the associations found in this study.

## Conclusions

Overall, the main findings of this study showed a high prevalence (7.2%) of CKD among adults who visited SIME clinics, which is comparable to the worldwide prevalence (9.1%) of CKD. Moreover, systolic blood pressure, sex and zone are the risk factors found to be associated with eGFR, yet further research needs to elucidate these associations and other risk factors that might be contributors of CKD in Ecuador. Although this study had its limitations, still can be considered as an incentive for Ecuador's main authorities to execute high quality population-based studies that accurately describe CKD in Ecuador and its provinces.

## Data availability statement

The data analyzed in this study is subject to the following licenses/restrictions: Due to HIPPA compliance records are not available to the public. Requests to access these datasets should be directed to leguiguren@ufl.edu.

## Ethics statement

The studies involving human participants were reviewed and approved by Ethics and Research Committee of Human subjects of Universidad San Francisco de Quito CEISH-USFQ (IE02-E158-2021-CEISH-USFQ) and by the University of Florida Institute of Review Board (IRB202101202). Written informed consent for participation was not required for this study in accordance with the national legislation and the institutional requirements.

## Author contributions

LE-J and JA: conceptualization, methodology, and writing—original draft preparation. LE-J and JM: data analysis, formal analysis, and data curation. LE-J, JM, JO, and JA: writing—review and editing. JA: supervision and project administration. All authors have read and agreed to the published version of the manuscript.

## Conflict of interest

The authors declare that the research was conducted in the absence of any commercial or financial relationships that could be construed as a potential conflict of interest.

## Publisher's note

All claims expressed in this article are solely those of the authors and do not necessarily represent those of their affiliated organizations, or those of the publisher, the editors and the reviewers. Any product that may be evaluated in this article, or claim that may be made by its manufacturer, is not guaranteed or endorsed by the publisher.
